# Headache characteristics in acromegaly: Only a secondary disorder?

**DOI:** 10.1111/head.14927

**Published:** 2025-03-13

**Authors:** Giada Giuliani, Denise Costa, Chiara Pellicano, Patrizia Gargiulo, Camilla Virili, Vittorio Di Piero, Marta Altieri

**Affiliations:** ^1^ Department of Human Neuroscience Sapienza University of Rome Rome Italy; ^2^ Department of Experimental Medicine, Endocrinology‐Pituitary Disease Sapienza University of Rome Rome Italy; ^3^ Department of Translational and Precision Medicine Sapienza University of Rome Rome Italy; ^4^ Department of Medico‐Surgical Sciences and Biotechnologies Sapienza University of Rome Latina Italy; ^5^ University Consortium for Adaptive Disorders and Head Pain (UCADH) Pavia Italy

**Keywords:** acromegaly, diagnosis, migraine, pituitary adenoma, secondary headache

## Abstract

**Objective:**

To investigate the characteristics and nature of headache in a population of patients with acromegaly.

**Background:**

Headache is frequently described by patients with pituitary adenomas. Although it is mainly considered a secondary disorder, it can persist despite effective therapy for pituitary disease. A proper description of headache according to the subtype of pituitary adenoma is not available in literature. In this light, we aimed to analyze headache characteristics in a population of patients with acromegaly.

**Methods:**

In this registry‐based retrospective cohort study, headache features were collected through a structured telephone interview. The clinical picture of each patient was classified according to the third edition of the *International Classification of Headache Disorders* criteria. We carefully investigated the time course and the relationship of headache with acromegaly.

**Results:**

Out of 39 enrolled patients, 27 (69%) reported headache. Six patients (15%) fulfilled secondary headache criteria, with complete headache resolution after acromegaly treatment. In all, 21 patients (54%) met the criteria for a primary headache: fourteen had episodic migraine, four had chronic migraine, and three had tension‐type headache. No trigeminal autonomic cephalalgias were observed. The presence of primary headache significantly reduced the time to diagnosis of acromegaly (mean [standard deviation] 2.1 [2.5] vs. 4.3 [3.5] years, *p* = 0.007). The occurrence of primary headache was similar in patients with macroadenoma compared to patients with microadenoma (14 [67%] vs. 7 [33%], χ^2^ = 0.591, *p* = 0.400), while its course was not significantly influenced either by the acromegaly treatment (*p* = 0.670) or the achievement of biochemical control (*p* = 0.490).

**Conclusion:**

Secondary headache was found only in a small percentage of our patients. Most of them had a primary headache with a high prevalence of migraine, suggesting that acromegaly might act as a trigger for this disorder. Considering the potentially disabling nature, primary headaches in patients with acromegaly require careful evaluation and personalized management.

## Plain Language Summary

Headache is commonly reported by patients with pituitary adenomas, but we do not know much about how the conditions are related. We wanted to understand the relationship between headache and acromegaly, so we studied how patients with acromegaly are affected by headache. We learned that many patients with acromegaly have a primary headache disorder that is not resolved once the adenoma has been addressed, so these patients may require careful evaluation and personalized management.

**Abbreviations**
GH: growth hormone
IGF‐1: insulin‐like growth factor‐1
IQR: interquartile range
NO: nitric oxide
SD: standard deviation
SSA: somatostatin analogs

## BACKGROUND

Headache is frequently reported by patients with different types of pituitary adenomas, with a prevalence ranging from 33% to 72%.^1^, ^2^ It can be the initial manifestation and have variable degrees of severity, sometimes playing an essential role in reaching the correct diagnosis.^3^ Headache associated with pituitary disease is considered a secondary disorder and no specific pituitary adenoma seems to cause a particular headache syndrome. Nevertheless, it can persist despite surgical removal of the adenoma or the achievement of biochemical control. Its pathophysiology is quite complex and is only partially justified by mechanical factors, such as tumor size, dural stretch, and cavernous sinus invasion.^4^ Indeed, neuroendocrine aspects and hypothalamic–pituitary axis disturbances might be crucially involved in its development. The effect of treatments for underlying disease on headache has received considerable attention. It is well known that only a small percentage of patients experience a significant reduction in headache after neurosurgery^5^; also, medical therapies appear to make limited contributions.^6^ At present, no proper characterization and description of headache according to pituitary adenoma subtype is available in the literature.^7^ Acromegaly is a rare disease caused by excessive production of growth hormone (GH), mostly by a pituitary adenoma, and up to 60% of affected patients complain of headache.^8^ The use of highly effective therapeutic strategies in restoring normal hormone levels, including long‐acting somatostatin analogs (SSA), does not seem to significantly modify this symptom.^9^ Unlike cardiovascular comorbidities, headache is usually not addressed during acromegaly follow‐up, even though it can have a negative impact on patients’ quality of life. The complex biochemical alterations induced by acromegaly could play a role in the development of headache, which might not be only a secondary disorder to the pituitary condition. In this light, we decided to analyze the headache characteristics in a population of patients with acromegaly.

## METHODS

This was a registry‐based retrospective cohort study designed to detect headache occurrence and course in patients affected by acromegaly, regularly followed in the Center for the Management of Pituitary Diseases, Department of Experimental Medicine Endocrinology, at Sapienza University of Rome, Italy.

The Regional Ethics Committee of Lazio (Area 1), Italy, approved the study (Ref 6817 Prot 0640/2022); all patients provided written informed consent in accordance with the Declaration of Helsinki.

### Acromegaly assessment

Demographic and clinical data about the acromegaly diagnosis, symptoms, and signs were obtained from medical records between 2000 and 2023. The inclusion criteria were as follows: a confirmed diagnosis of acromegaly, aged ≥18 years, written informed consent, being subjected to acromegaly treatment, and a minimum follow‐up period of 1 year. Patients were enrolled regardless of the severity of the disease and clinical manifestations at the time of onset. The only exclusion criterion was being aged <18 years. The diagnosis of acromegaly was made according to the guidelines of the Endocrine Society.^10^ In patients with elevated or equivocal serum insulin‐like growth factor‐1 (IGF‐1) concentrations, confirmation of the diagnosis was made by finding a lack of suppression of GH to <0.4 μg/L, following documented hyperglycemia 2 h after 75 g of oral glucose load. Biochemical disease control was defined by determining age, IGF‐1, and GH levels (expressed in ng/mL).

The diagnostic delay was calculated as the elapsed time between the date of the first reported comorbidity and the date of acromegaly diagnosis.^11^ The date of diagnosis was defined as the first specialized healthcare visit or admission with acromegaly diagnosis while the date of onset of the first comorbidity was defined as the first registration of any predefined comorbidity from medical records.

Follow‐up was performed according to international guidelines.^12^, ^13^ In our population, the duration of the monitoring period varied, reaching up to 20 years in some cases. Patients underwent clinical and biochemical examinations every 6 months. Instrumental tests for the evaluation of major comorbidities associated with acromegaly were performed at different intervals, often tailored to individual needs. Gadolinium‐enhanced pituitary magnetic resonance imaging was obtained in all patients at diagnosis using high‐quality, high‐resolution equipment, such as 1.5‐ or 3‐T scanners, where available, including T1‐ and T2‐weighted fast spin echo sequences, with coronal and sagittal planes in 2–3 mm slice thickness with no or minimal spacing. Information about invasion into surrounding structures and adenoma dimensions was recorded.^14^, ^15^ Pituitary tumor size was calculated by measuring adenoma diameters: according to the largest tumor diameter, tumors were classified as microadenomas (diameter <10 mm) or macroadenomas (>10 mm). All patients received treatments for acromegaly (neurosurgery, radiotherapy, medical therapy, alone or in combination). Surgical therapy and SSA were recommended as first‐line treatments. Surgery, typically performed using a transsphenoidal approach, was generally the primary treatment of choice. Outcomes depended on preoperative tumor size, extension, and GH levels; in specific cases, surgical tumor debulking was performed before initiating medical therapy.^16^ Patients with active acromegaly and partial response or resistance to first‐generation SSA were treated with second‐line drugs, represented by long‐acting somatostatin receptor ligands or GH receptor antagonist. Full response to first‐generation SSA was defined as ameliorating patients’ clinical signs and symptoms and normalizing GH and IGF‐1 levels. Partial response to first‐generation SSA was defined in patients who did not achieve the normalization of GH and IGF‐I, but with a significant decrease of GH and IGF‐I levels (at least by 50% in comparison to pre‐treatment levels) and/or in patients with a tumor shrinkage of at least 20%. Poor response or resistance to first‐generation SSA was defined in patients without a significant decrease of GH and IGF‐I levels (<50% in comparison to pre‐treatment levels) and the absence of tumor shrinkage.^17^

### Neurological evaluation

Patients affected by acromegaly, regularly subjected to laboratory, clinical, and radiological follow‐up, were considered eligible. They were examined by a neurologist trained in headache to assess their personal and family history of headache; data about the clinical features of headache were collected through a structured telephone interview, and an ad hoc questionnaire was completed.

The age of onset and duration of headache were investigated. The following pain characteristics were carefully collected: side, quality, and severity. Using a numeric rating scale, patients had to rate the intensity from 0 (“no pain”) to 10 (“worst possible pain”).^18^ The attack duration, frequency, and associated symptoms were described. The use of acute medications for headache attacks and the number of pills taken monthly were recorded. For each patient, the temporal relationship between headache onset and acromegaly diagnosis was analyzed, also focusing on tumor size and invasion of surrounding structures. In addition, the effect of acromegaly treatments on headache was carefully considered.

Finally, based on the clinical phenotype and course, we classified the headache of each patient according to the criteria of the third edition of the *International Classification of Headache Disorders*.^19^

### Statistical analysis

For the statistical analysis JASP Team (2024) version 0.17.2.1 software was used. There were no missing data. No statistical power calculation was conducted prior to the study and the sample size was based on the available data. After evaluation of distributions using Shapiro–Wilk test, continuous variables are expressed as the median and interquartile range (IQR) or as the mean and standard deviation (SD). Categorical variables are expressed as the absolute frequency and percentage (%). The Mann–Whitney test was used to evaluate differences in continuous/discrete variables with skewed distributions. The chi‐square test or Fisher's exact test was used to evaluate differences between categorical variables. All analyses with two‐tailed *p* < 0.05 were considered statistically significant.

## RESULTS

We enrolled 39 patients, of whom 24 were female (62%) and 15 were male (38%). The mean (SD) age was 55.8 (14.1) years, and the median (IQR) duration of acromegaly was 15.0 (12.0–24.0) years. In all, 28 patients (72%) had a diagnosis of macroadenoma, 11 patients (28%) had a diagnosis of microadenoma, and 12 patients (31%) had cavernous sinus invasion. Biochemical control of the disease was achieved in 29 (74%) patients and was maintained during the subsequent follow‐up. In all, 14 patients (36%) were radically treated with surgical therapy and 17 patients (44%) were treated with SSA, preceded by surgery in 13 patients who were taking first‐generation analogs. Ten patients (26%) had partial response or resistance to first‐generation SSA: nine of them (23%) were treated with a GH antagonist (pegvisomant).

Headache was present in 27 patients (69%): of these, 20 (51%) were biochemically controlled (Table 1). The achievement of biochemical control did not significantly influence the presence of headache (Fisher's exact test >0.999, *p* = 0.640).

**TABLE 1:** Biochemical control, acromegaly treatment and headache occurrence.

Patient number	Biochemical control	Acromegaly treatment	Headache
1	+	NCH	+
2	−	NCH*	+
3	+	NCH + SSA I	+
4	+	NCH+ SSA I	+
5	+	NCH	−
6	+	NCH + SSA I	+
7	+	NCH	+
8	−	NCH + SSA I/PEG+SSA II	−
9	+	NCH + SSA I	**+** ^**a**^
10	+	NCH + RT + SSA I + PEG	+
11	+	SSA I	+
12	−	NCH	+
13	−	NCH + SSA I + PEG	+
14	+	NCH + SSA I + PEG	**+** ^**a**^
15	+	NCH + SSA I	−
16	−	NCH + PEG	−
17	−	NCH + SSA I + PEG	+
18	+	NCH + SSA I	−
19	−	NCH + SSA I	+
20	+	NCH + SSA I+ PEG	+
21	+	NCH	+
22	+	NCH + SSA I	+
23	+	NCH	+
24	+	NCH	+
25	+	NCH*	+
26	+	NCH	−
27	−	NCH + SSA I+PEG+SSA II	**+** ^**b**^
28	+	NCH + RT+ SSA I	**+** ^**a**^
29	+	NCH	+
30	+	SSA I + PEG +NCH	**+** ^**a**^
31	+	NCH + SSA I	−
32	+	SSA I + PEG	**+** ^**b**^
33	−	NCH + PEG	−
34	+	SSA I	−
35	−	SSA I + PEG	+
36	+	NCH	−
37	+	NCH	−
38	+	SSA I	+
39	+	NCH + SSA I + PEG	−

*Note*: Six patients reported headache resolution after acromegaly treatment: four of them after surgery (a) and two after medical therapy (b). The bold values indicate the 6 patients with secondary headache in whom the headache disappeared after surgical (a) or medical (b) therapy.

Abbreviations: NCH, neurosurgery; PEG, pegvisomant; RT, radiotherapy; SSA I, somatostatin analogs (first generation); SSA II, somatostatin analogs (second generation).

* In two patients, first‐generation somatostatin analogs were recently introduced into treatment, long after the surgical procedure, due to a purely biochemical recurrence of the disease, with no impact on headache.

### Primary headache

A total of 21 patients (54%) presented a clinical picture consistent with a primary headache. Of these, 12 were female (57%), nine were male (43%), and the mean (SD) age was 51.5 (13.8) years. The mean (SD) age of headache onset was 25.0 (10.0) years, and the median (IQR) duration of disease was 26.0 (15.0–31.0) years. Of the 21 patients, 12 (57%) had a family history of headache.

The headache characteristics are summarized in Table 2. The mean (SD) number of headache days per month was 6.2 (9.1), and the median (IQR) duration of attack was 18.0 (12.0–24.0) h. Pain was preferentially located in the orbital, retro‐orbital, and frontal regions; it was unilateral in six patients (29%) and bilateral in 15 (71%). Regarding pain quality, 10 patients (48%) described it as throbbing and pulsating, while 11 (52%) described it as dull or squeezing. Based on the numeric rating scale, the median (IQR) intensity was 7.0 (6.0–8.0). Physical activity aggravated headache in 13 patients (62%). Photophobia and phonophobia during attacks were the most common accompanying symptoms (86%), while nausea, associated with vomiting in two cases, was present in eight patients (38%). No cranial autonomic symptoms were observed. Two patients reported visual disturbances immediately before the onset of headache, characterized by the gradual development of luminous phenomena and light flashes, for a maximum duration of 30 min.

**TABLE 2:** Headache characteristics in patients with acromegaly and primary headache.

Patient number	Sex	Age, years	Age of HO, years	Age of AD, years	MHDs	Duration, h	Unilateral pain	Severity, NRS score	Pulsating quality	APH	Photo/phono	Nausea	AM	MAMs	Improving with AT
1	F	26	6	25	3	12	−	7	−	+	+	−	+	5	+
2	F	31	20	27	2	12	−	7	+	+	+	−	+	2	+^c^
3	M	35	20	27	1	12	−	5	+	−	+	−	−	0	−
4	F	35	20	17	<1	6	+	6	−	+	+	−	+	0	−
5	M	41	30	38	3	36	−	8	+	−	+	−	+	0	−
6	F	42	16	39	1	12	−	5	−	−	−	−	+	1	+
7	F	45	30	29	1	6	−	7	+	+	+	−	+	1	−
8	F	47	16	40	4	24	+	8	−	−	+	+	+	2	−
9	F	47	20	35	28	24	+	8	+	+	+	−	−	0	+
10	F	48	28	38	25	24	+	5	−	+	+^a^	+^b^	−	0	−
11	F	52	30	38	2	24	−	7	+	+	+	+	+	1	+
12	M	55	20	52	1	12	−	7	+	−	+	+	+	<1	+
13	M	57	30	43	1	6	−	6	−	−	−	−	+	1	−
14	M	59	50	40	3	6	−	8	−	+	+	−	+	3	−
15	F	60	20	34	15	24	−	7	−	+	+	+	+	18	−
16	M	60	40	46	2	12	−	7	−	+	+	−	+	2	−
17	F	62	30	46	3	24	−	7	+	+	+	+	+	3	+
18	M	62	11	21	3	24	+	6	+	+	+	+^b^	+	3	−
19	M	66	36	40	3	12	−	9	+	−	+	−	+	5	−
20	M	73	30	60	1	12	−	3	−	−	−	−	−	0	+
21	F	79	20	75	28	24	+	8	−	+	+	+	+	10	−

Abbreviations: AD, acromegaly diagnosis; AM, acute medication (nonsteroidal anti‐inflammatory drugs or acetaminophen); APH, aggravated by physical activities; AT, acromegaly treatment; F, female; HO, headache onset; M, male; MAMs, monthly acute medications; MHDs, monthly headache days; NRS, numeric rating scale; phono, phonophobia; photo, photophobia.

^a^ Only phonophobia.

^b^ Nausea and vomiting.

^c^ Disappearance of visual aura after surgery.

In all, 17 patients (81%) used acute medications for attacks (nonsteroidal anti‐inflammatory drugs or acetaminophen), and the mean (SD) number of pills taken per month was 2.7 (4.2). No patient used migraine‐specific drugs such as triptans. A patient in their seventies underwent peripheral nerve block of the greater occipital nerve with anesthetic several decades ago; no other prophylactic treatment was reported.

Out of 21 patients, 18 (86%) met the criteria for a diagnosis of migraine: 14 (67%) were affected by episodic migraine, while four (19%) had chronic migraine. Of these, two patients had migraine with typical aura. Tension‐type headache was present in three patients (14%). No trigeminal autonomic cephalalgias were observed.

### Secondary headache

**FIGURE 1:**
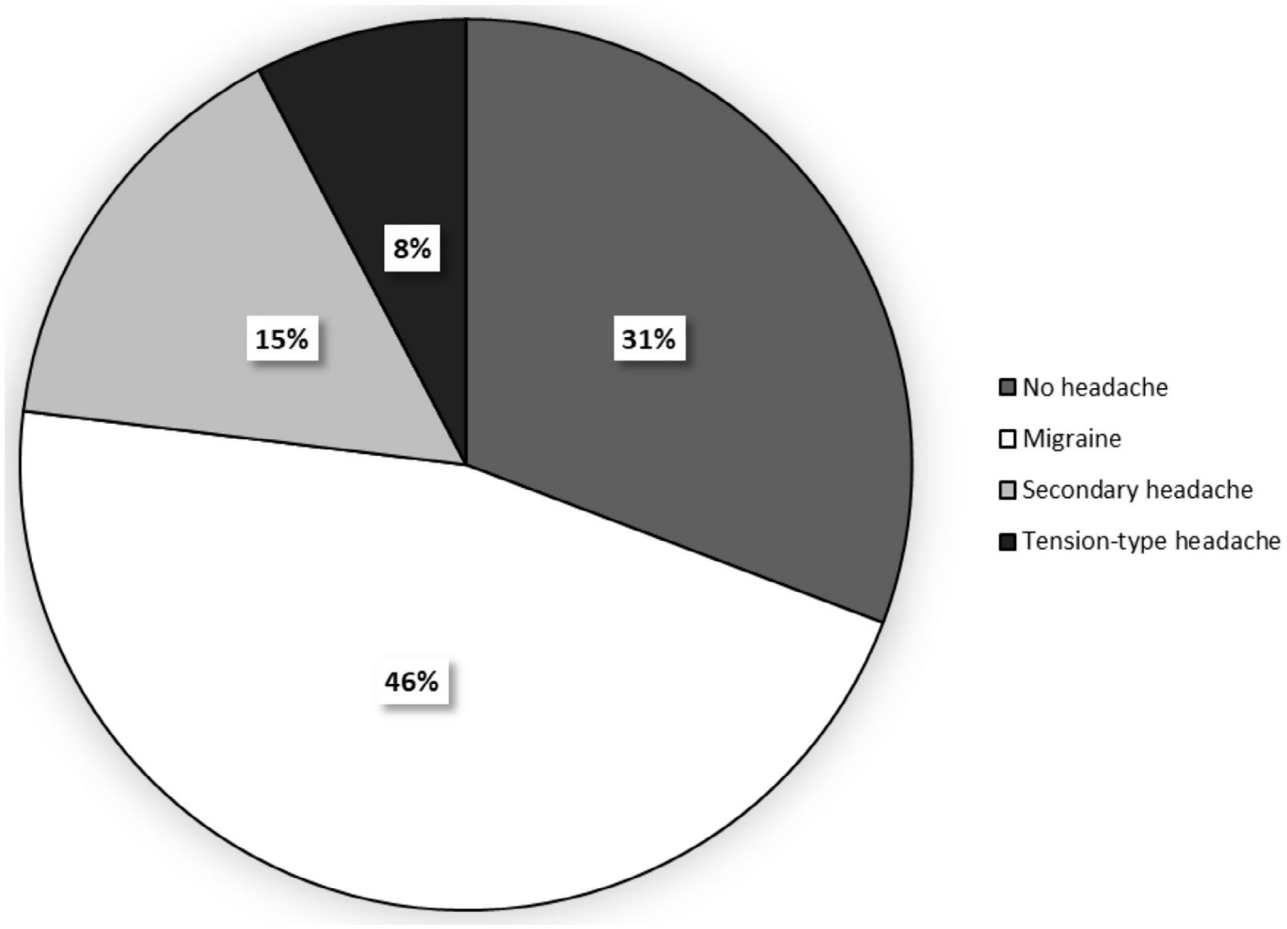
Headache disorders in patients with acromegaly.

Six patients (15%) presented a picture consistent with secondary headache, fulfilling the criteria for “7.4.3 Headache attributed to hypothalamic or pituitary hyper‐ or hyposecretion” of the third edition of the *International Classification of Headache Disorders*.^17^ These patients described a feeling of bilateral pressure in the orbital or supraorbital region, with moderate or severe intensity, without specific accompanying symptoms. In all of them, headache represented the initial manifestation of the pituitary adenoma with complete remission after treatment: four patients reported improvement after surgery and two after medical treatment. Five of them presented with a macroadenoma, with the invasion of the cavernous sinus in two cases, without other neurological signs. At the time of neurological evaluation, all patients except one had achieved biochemical control of the disease and none of them reported headaches (Figure 1).

## ACROMEGALY AND PRIMARY HEADACHE

### Time to diagnosis

**FIGURE 2:**
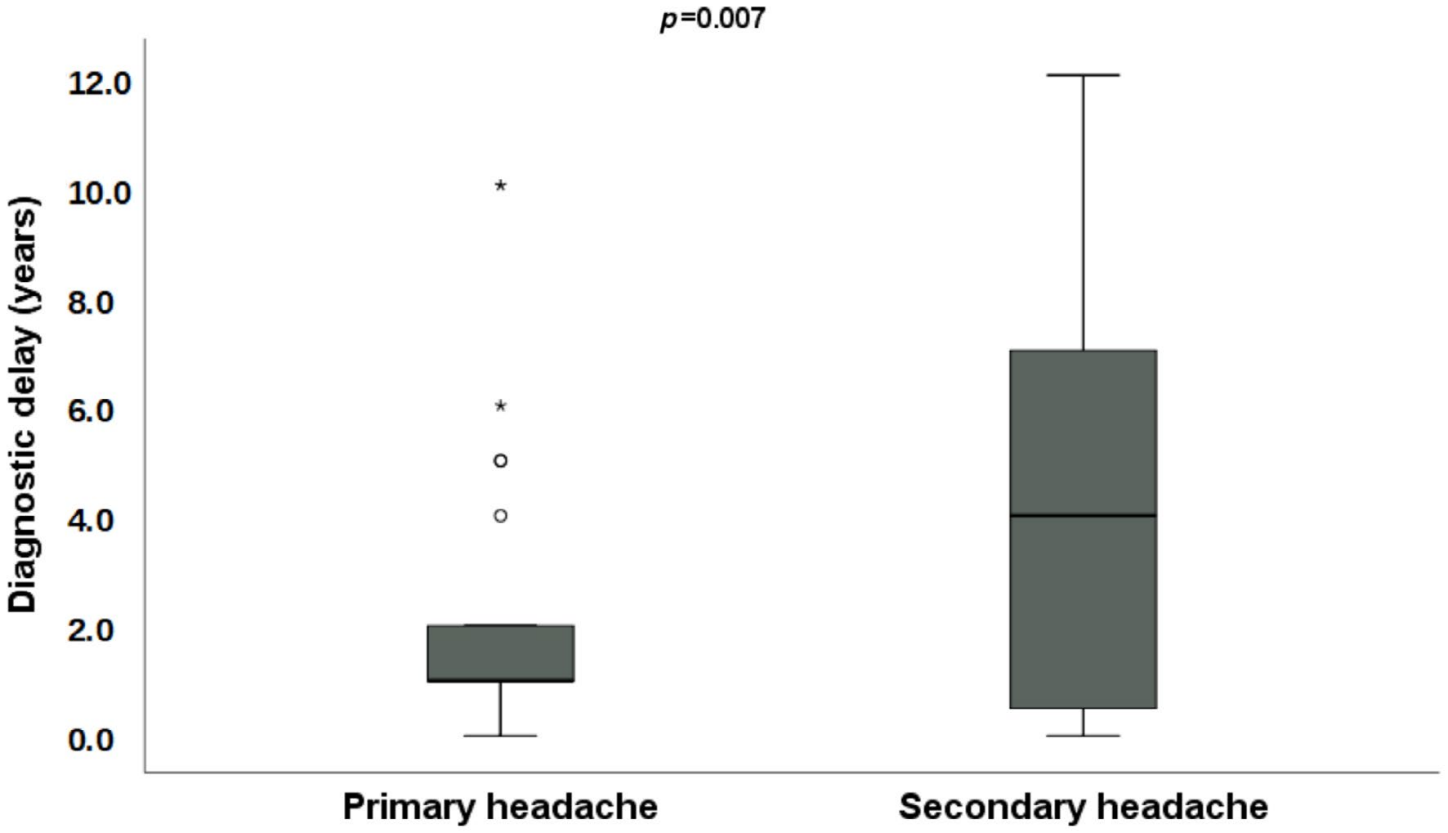
Time to acromegaly diagnosis was significantly lower in patients with primary headache. [Colour figure can be viewed at wileyonlinelibrary.com]

The median (IQR) duration of acromegaly was 22.0 (14.0–32.7) years in patients without headache and 15.0 (7.0–18.0) years in patients with primary headache (*p* = 0.020). The time to diagnosis was significantly different between the two groups (Figure 2): the mean (SD) diagnostic delay in patients with primary headache was 2.1 (2.5) years, while it was 4.3 (3.5) years in patients without a personal history of headache (*p* = 0.007).

### Tumor size and cavernous sinus invasion

Among the 21 patients with primary headache, 14 had macroadenomas (67%), and seven (33%) had microadenomas. Tumor size did not significantly influence headache presence (χ^2^ = 0.591, *p* = 0.400). Six patients (29%) presented with cavernous sinus invasion, described according to the Knosp classification.^20^ An association was not found between cavernous sinus invasion and headache (χ^2^ = 0.103, *p* = 0.740, Table 3).

**TABLE 3:** Cavernous sinus invasion and headache lateralization in patients with acromegaly and primary headache.

*N*	Cavernous sinus invasion^a^	Headache lateralization
1	0	Bilateral
2	1–2	Bilateral
3	0	Bilateral
4	0	Bilateral
5	0	Bilateral
6	4	Unilateral

^a^ Cavernous sinus invasion was defined according to the Knosp classification,^20^ used to estimate surgical remission after pituitary adenoma resection.

### Effect of acromegaly treatment

Acromegaly treatment did not significantly affect the course of primary headache (*p* = 0.670). Eight patients with primary headache (38%) reported a slight improvement of headache after acromegaly treatment without its complete resolution: among these, a woman affected by migraine with typical aura described the disappearance of only aura episodes after neurosurgery, with the persistence of migraine attacks without aura. Out of the 21 patients, 15 achieved biochemical control whereas six showed a partial response or resistance to first‐line therapies. Biochemical control did not significantly influence the presence of primary headache (*p* = 0.490).

## DISCUSSION

To our knowledge, this is the first study on headache occurrence in a population of patients with acromegaly. The detailed description of headache, with the analysis of its time course and relationship with pituitary disease, is based on the extended follow‐up period, which can reach up to 20 years. Headache was frequently reported by our patients, but it was the initial manifestation of acromegaly only in a small percentage of cases. Additionally, the involvement of the cavernous sinus did not cause the development of trigeminal autonomic cephalalgia‐like syndrome or other neurological signs.^21^ Hence, we can assert that secondary headache has only a limited role among the symptoms associated with acromegaly.

Interestingly, most of our patients fulfilled the criteria for primary headache: 46% of them were affected by migraine, with a higher prevalence than that of the general population.^22^ Overall, a family history of headache was present in 20 patients (51%). This suggests that patients with acromegaly may have a predisposition to migraine.^23^ The temporal course of migraine in our population appears to be poorly influenced by adenoma‐related factors, such as tumor size or cavernous sinus invasion. Some of our patients described an improvement in the frequency and intensity of attacks over the years, which was mainly related to age. Similar changes in the most bothersome symptoms and sensory hypersensitivity are commonly observed in patients with migraine with aging.^24^ A recent study analyzed plasma concentrations of calcitonin gene‐related peptide and pituitary adenylate cyclase‐activating peptide in patients with pituitary adenomas and reported higher values in patients with headache.^25^ It is well known that these peptides are involved in migraine pathophysiology, and their plasma levels are typically increased in migraine population.^26^ Keeping in mind these findings, headache seems to be not just a manifestation of acromegaly but rather a comorbidity.

Acromegaly is typically caused by a GH‐secreting pituitary adenoma that drives excess secretion of IGF‐1. Under normal conditions, these hormones ensure the proper endothelial function, by regulating proinflammatory cell recruitment and nitric oxide (NO) production.^27^ In turn, NO helps maintain vascular homeostasis and inhibits platelet aggregation. Excessive levels of IGF‐1 and GH are associated with endothelial progenitor cell dysregulation, altered release of NO, and increased oxidative stress.^28^, ^29^ Impaired anti‐inflammatory responses, such as a decreased production of interleukin‐10, have been also observed in patients with acromegaly, even after effective treatment.^30^ The systemic inflammatory state and endothelial dysfunction, partially independent of the normalization of hormonal levels and already considered responsible for increased cardiovascular risk in these patients, might also act as triggers for migraine.^31^ In this light, acromegaly might promote proinflammatory and pronociceptive changes in predisposed individuals, modifying the threshold for the occurrence of attacks and influencing the severity of the disease. Due to the complex interactions among these factors, headache could become a disabling comorbidity.

Acromegaly therapies showed only a limited effect on migraine course in our study. According to the literature, new drugs, such as second‐generation somatostatin receptor agonists, which are able to provide better hormonal control through greater affinity for the somatostatin receptor than first‐generation agents, seem to improve headache in only a small percentage of treated patients.^32^ It is possible that the different receptor‐binding profile could have superior and longer‐lasting analgesic effects than older treatments, with a consequent benefit on secondary headache. We might wonder whether more advantageous hormonal control and a more effective reduction in the inflammatory condition related to acromegaly might have a positive impact on migraine. However, although acromegaly might act as a triggering or aggravating element, its treatment could have only a partial effect on migraine course considering the complex nature of this primary headache. Future studies are needed to explore the full potential of these new drugs.

Our study shows the double role of primary headache in patients with acromegaly. Promoting the evaluation through neuroimaging, it can allow the early detection of acromegaly and helps to direct the patient towards appropriate treatment.

On the other hand, primary headache is frequently overlooked and undertreated with a potential negative effect on life quality. We, therefore, suggest that headache assessment should become an integral part of the follow‐up for acromegaly: patients who continue to experience headaches despite effective acromegaly treatment should be referred for a neurological evaluation. In this population, it might be interesting to explore the benefit of preventive options typically used for primary headaches while the availability of new anti‐migraine drugs might represent a further strategy to reduce disability. A better understanding of the pathophysiological mechanisms of headache in acromegaly through future studies is essential to improve patient management and to guide medication choice.

The main limitation of the present study is its retrospective design: our data were collected through patients’ interviews and the risk of recall bias cannot be excluded. Another limitation is the small sample size, which did not allow us to carry out more detailed analysis and limited results’ generalizability. However, it should not be forgotten that acromegaly is a rare disease, and this is the experience of a single center. A similar number of patients, considering the extended follow‐up period, is not insignificant. Still, future prospective and multicenter studies are necessary to increase sample size and to confirm our results.

## CONCLUSIONS

Contrary to expectation, secondary headache is a manifestation of acromegaly only in a small percentage of patients. It shows a close temporal relationship with pituitary disease and completely disappears after effective acromegaly treatment. Interestingly, we observed a high prevalence of migraine, along with some cases of tension‐type headache, in patients with acromegaly. When headache persists despite effective therapy, it may represent a comorbidity associated with acromegaly rather than a manifestation of it. Patients who continue to experience headache despite acromegaly treatment should be referred for a neurological evaluation and proper assessment. Acute and preventive options typically used for primary headache disorders might be useful to reduce headache burden in these patients. However, despite the negative effect on quality of life, primary headache in patients with acromegaly may have a positive aspect, as it could lead to more frequent neuroimaging and ultimately facilitate an earlier detection of the endocrine disease.

## AUTHOR CONTRIBUTIONS

**Giada Giuliani:** Conceptualization; data curation; investigation; writing – original draft; writing – review and editing. **Denise Costa:** Conceptualization; investigation; writing – review and editing. **Chiara Pellicano:** Formal analysis; writing – review and editing. **Patrizia Gargiulo:** Supervision; validation. **Camilla Virili:** Writing – review and editing. **Vittorio Di Piero:** Validation; visualization. **Marta Altieri:** Supervision; writing – review and editing.

## FUNDING INFORMATION

The author(s) received no financial support for the research, authorship, and/or publication of this article.

## CONFLICT OF INTEREST STATEMENT

**Giada Giuliani, Denise Costa, Chiara Pellicano, Patrizia Gargiulo, Camilla Virili, Vittorio Di Piero**, and **Marta Altieri** have no conflicts of interest to declare.

## ETHICS APPROVAL AND CONSENT TO PARTICIPATE

This study complied with the Declaration of Helsinki; the Regional Ethics Committee of Lazio (Area 1), Italy, reviewed and approved the research protocol (Ref 6817 Prot 0640/2022). Written informed consent for participation was obtained from the subjects.

## Data Availability

The datasets used and/or analyzed during the current study are included in this manuscript. Further information is available from the corresponding author upon reasonable request.
